# Transcriptional modulation of inflammation, and aging in Indian obese adults following a 12-week yoga-based lifestyle intervention: A randomized controlled trial

**DOI:** 10.3389/fmed.2022.898293

**Published:** 2022-08-08

**Authors:** Piyush Sharma, Raj Kumar Yadav, Rajesh Khadgawat, Rima Dada

**Affiliations:** ^1^Integral Health and Wellness Clinic, Department of Physiology, All India Institute of Medical Sciences, New Delhi, India; ^2^Department of Endocrinology, Metabolism and Diabetes, All India Institute of Medical Sciences, New Delhi, India; ^3^Department of Anatomy, All India Institute of Medical Sciences, New Delhi, India

**Keywords:** aging, inflammation, obesity, oxidative stress, yoga

## Abstract

**Introduction:**

Obesity is one of the major global problems in today's world, both in children, and the adult age group. Current evidence suggests obesity alters the expression of various genes related to oxidative stress, inflammation, and aging. In recent times complementary therapy like yoga-based lifestyle intervention (YBLI) is used as an adjunct therapy to modern medicine. This study examines the efficacy of 12 weeks of yoga-based lifestyle intervention with standard care (SC) on the expression of genes related to oxidative stress, inflammation, and aging in obese adults.

**Methods:**

This was a two-arm parallel randomized control trial implemented at Integral Health Clinic (IHC), an outpatient facility that regularly conducted YBLI programs for the prevention of lifestyle diseases like obesity and diabetes in the Department of Physiology, All India Institute of Medical Sciences (AIIMS), New Delhi. Blood samples at baseline and weeks 2,4, and 12 were collected from 72 adults (male *n* = 21; female *n* = 51) of age 20–45 years with a body–mass index (BMI) of 25–35 kg/m^2^ who were randomized to receive either a 12-week SC (*n* = 36) or YBLI (*n* = 36). SC included recommendations for the management of obesity as per Indian guidelines including a low-calorie individualized diet and physical activity. *Asana* (physical postures), *pranayama* (breathing exercises), and meditation were all part of the YBLI. Primary outcomes were relative fold change in the expression of genes associated with oxidative stress [Nuclear factor-kappa B (NF-Kappa B)], inflammation [Tumor necrosis factor-α (TNFα), interleukin-6 (IL-6)], and aging [human telomerase reverse transcriptase (TERT)] in peripheral blood mononuclear cells between the two groups at week-12.

**Results:**

There were no significant changes in fold change of TERT, IL-6, and NF-kappa B between the groups at week 12. The relative fold change of TERT was significantly greater in the YBLI group (*p* = <0.0001) vs the SC group at 2 weeks. The relative fold change of TNF α was significantly lower at week 12 in YBLI though the change was not continuous and reliable. Within both groups, TERT expression was significantly increased at week 2 though the change was greater in the YBLI group (*p* < 0.0001). TNF α gene expression was significantly lower at weeks 2 and 4, compared to baseline level, in the SC group but it increased at week 12.

**Conclusion:**

The results while did not confirm our hypothesis, are important to share with the scientific society, to be able to improve prospective study designs and find optimal time/intervention/biological marker settings for this highly important scientific field. The results are suggestive of a positive impact of YBLI and SC on the fold change of aging-related TERT gene in obesity, though the benefit was not evident till week 12. However, the results should be evaluated with caution and in light of other published studies. To better understand the positive effects of YBLI on oxidative stress, inflammation, and aging-related gene expression in obesity, larger studies are recommended.

## Introduction

Obesity is a low-grade chronic inflammatory condition that is a risk factor for many chronic diseases such as cardiovascular diseases, metabolic syndrome and cancers, etc (1). In 2016, globally more than 1.9 billion adults, 18 years and older, were overweight. Of these, over 650 million were obese (2). Obesity is a modern developed world disease that arises because of the rapid increase in economic growth, easy availability of inexpensive nutrient-poor food and urbanization, etc. Various lifestyle habits like lack of physical activity, imbalanced diet, irregular sleeping patterns, screen time, and stress might have been associated with weight gain problems in adults (3, 4). Human stressors are the key component that can elevate the level of stress hormones like catecholamines and cortisol. This is mediated by the neuroendocrine and sympathetic nervous systems—the primary immune and physiological mediators. Lack of sleep modulates our neuroendocrine system which induces the activation of stress-responsive genes (5). The BMI cut-off for obesity is different for Asian Indian adults from that of the US population. As per Asian Indian criteria, BMI > 25 kg/m2 is considered obese whereas as per the WHO criteria, > 25 kg/m2 is overweight, and >30 kg/m^2^ is obese (1, 6). In the Indian population, it has been reported that cardiovascular diseases and type 2 diabetes mellitus-like disorders have been associated with lower BMI values as compared to age-matched white Caucasians (6).

Obesity is a global problem characterized by an increase in body weight and excessive accumulation of adipose tissue (7) accompanied by various adverse health conditions (8, 9). These conditions are linked with increased oxidative stress levels as well as chronic inflammation that obesity provokes (10, 11).

Adipose tissue functions as an endocrine tissue and also helps in maintaining energy homeostasis. The adipose tissue consists mainly of adipocytes and other immune cells (12). These adipocytes secrete an array of cytokines like adipokines which modulate the molecular signals that may be causing metabolic complications (13). Adipokines in physiological conditions induce the reactive oxygen species which increases oxidative stress (14). Increased oxidative stress activates the inflammation status by increasing the level of various inflammatory cytokines like tumor necrosis factor α (TNF-α), Interleukin−6 (IL-6), and interleukin (IL)-1β, etc (15).

At the molecular level obesity might alter the expression of various stress and inflammatory genes like TNF-α and IL-6 (16). It is also seen that the Nuclear Factor kappa-B (NF-kappa B) is activated by oxidative stress and is associated with all types of inflammation responses linked with obesity (17). Increased gene expression of inflammatory markers like TNF-α and IL-6 has been associated with immunosenescence (aging-triggered immune dysfunction) conditions in obese elderly adults (18). Obesity is also associated with cellular aging due to the shortening of chromosomal ends. These chromosomal ends are usually protected by the short stretches of DNA segments (TTAGGG) known as telomeric region, These telomere segments are synthesized by telomerase enzyme, whose catalytic subunit gene is hTERT (human telomerase reverse transcriptase) (19).

“*Yoga*” means union of our individual consciousness with the Universal Divine Consciousness in a super-conscious state known as *Samadhi* (20). Yoga brings balance and health to the physical, mental, emotional, and spiritual dimensions of an individual. Yoga encompasses mental and physical discipline to help in personal transformation that leads to perfect health as envisioned by WHO. YBLI is an integrated package of theory and practice sessions with yoga at its core, and embraces all dimensions as listed above, and aims to make it a “lifestyle” rather than a “momentary practice”. A typical day starts with a set of *asanas* (physical postures), *pranayama* (breathing exercises), *dhyana* (meditation) conducted by a trained yoga instructor for approximately 1 h to set a positive start of the day. The participants are then pulled in a mind engaging activity that orients them toward a yoga-based healthy lifestyle. This is delivered in the form of discussions/lectures on principles of yoga, yogic techniques, stress management, healthy diet, and eliminating elements of negativity (substance abuse etc.). The intervention is delivered to a group of six to eight participants at a time, allowing them ample time to interact with the experts (medical doctors, yoga experts, and dietitians). The YBLI used in this study has been pretested and published previously (21–30).

Lifestyle interventions like *Sudarshan kriya* feature cyclical breathing patterns that range from slow and relaxing to quick and energizing. *Sudarshan kriya* involves three separate rhythms of breathing. Initially, it is started with *Ujjayi Pranayama* (long and deep breaths with constriction at the base of the throat) and then, *Bhastrika* (fast and forceful breaths through the nose along with arm movements). *Sudarshan kriya* seems to have a positive impact on oxidative stress and cellular aging at the genetic level by showing the trend of higher expression of antioxidant genes like glutathione peroxidase as well as the aging-related hTERT gene, though it was not statistically significant (31).

Yoga-based therapy improves the molecular signatures of genes associated with metabolism, oxidative phosphorylation, generation of reactive oxygen species, and oxidative stress at the transcriptomic level (32) authors did not mention the mechanisms behind yoga while impacting metabolism and oxidative stress, etc. However, previously published articles showed that yoga meditation might regulate the rate of metabolism by improving digestion, circulation, and muscle flexibility. Yoga stimulates and strengthens our endocrine organs, leading to favorable metabolic effects (21). Yoga intervention also proved to be beneficial in maintaining the balance between reactive oxygen species levels and the antioxidant system. Yoga balances the over-activation of hypothalamic-pituitary–adrenal axis and sympathoadrenalmedullary axis during stress. Importantly, *pranayama* emphasizes long exhalation and conscious slow breathing that positively modulates the parasympathetic nervous system (33, 34). Yoga-meditation influences different areas of the brain, specifically on the right side of the brain, which might influence the neuroendocrinological axis (30, 35).

Another study showed that *Sudarshan Kriya and Related Practices* (an advanced form of rhythmic, cyclical breathing with slow, medium, and fast cycles) have a rapid and significantly effect on gene expression in peripheral blood mononuclear cells (36). Mindfulness meditation significantly reduced the pro-inflammatory gene expression in the peripheral blood mononuclear cells of meditators as compared to healthy control (37). A randomized control trial showed that 8 weeks of *Kirtan Kriya Meditation* a mediation technique used in *Kundalini yoga*, is intended to stimulate the senses and parts of the brain. It involves a combination of chanting and finger movements. *Kirtan Kriya Meditation* might reverse the pattern of increased NF-kappa B-related transcription of pro-inflammatory cytokines in dementia caregivers (38). The transcriptomic level of NF-kappa B was significantly reduced in breast cancer survivors after 12 weeks of Iyenger Yoga intervention (39). Among the breast cancer survivors, Mind-body therapy like Tai Chi significantly reduced the gene expression of TNF-α, a similar trend has been shown by the IL-6 but it was not statistically significant (40). Yoga practice is shown to increase the expression of the hTERT gene restraining the cellular aging process in hypertensive patients (41). YBLI of 8 weeks has significantly reduced the expression of TNF-α and IL-6 genes in rheumatoid arthritis patients (42).

YBLI seems to be a potential intervention that benefits people across indications, e.g., depression, obesity, hypertension, type 2 diabetes, and cancer (43, 44).

We have demonstrated a significant reduction in stress, inflammation, and oxidative stress biomarkers in blood in obese individuals (24, 45). Recently, we have also reported the positive impact of YBLI on markers of cellular aging (telomere length and telomerase level) in obese individuals (29).

However, there is a paucity of literature regarding randomized controlled trials conducted on the YBLI and SC in obesity to observe transcriptomic changes.

Therefore, the primary objective of this RCT was to compare the effectiveness of a 12-weeks YBLI and SC in triggering relative changes, if any, in oxidative stress, inflammation, and aging-related genes in Indian obese individuals.

## Materials and methods

### Sample size calculation

The main project (study) was conceptualized to evaluate the effects of Yoga-based lifestyle intervention with standard care on telomere length, telomerase level, and mRNA fold changes of genes related to oxidative stress, inflammation, and aging. There were no published studies at the time of conception of this study using such intervention in the intended population, i.e., Indian adults with obesity. Therefore, sample size was calculated using the data from the closest matching studies. Sample size calculation was done using the co-primary endpoints, i.e., telomere length and telomerase level as in our recently published article (29).

For the present study, we referred to three studies (31, 37, 46). For calculating the effect size, we referred (31). Even though the study population was different in terms of age, gender, intervention, and outcome measures, these were the closest data available. The calculations were done using a *t*-test (independent means of two groups), a priori: Compute required sample size analysis approach in G^*^ Power (Version 3.1.9.2), and the inputs were the effect size (0.8), power (1-β) of the study (80%), and type I (α) error (5%). Based on the calculated sample size of this study, the biostatistician suggested enrolling at least 30 individuals in each of the two groups; considering the dropout rate of 10–20 % (47) a total of 72 participants (36 in each of the SC and YBLI) were to be recruited.

#### Randomization

All the participants of the study (*n* = 72) were randomly allocated to one of the two intervention groups to receive SC or YBLI. The random number list was created using the software QUERY Advisor (version 7.0, United States) employing a block randomization protocol with a block size of 8 using a computer-generated alphanumeric random code by a biostatistician who was not involved in the study. Eligible participants were assigned to the YBLI group or the SC group according to the randomization list. To ensure allocation was concealed, an independent investigator unrelated to the study placed each allocation sequence in separate envelopes, and immediately after the initial assessment, eligible participants opened the allocation sequence in a sealed envelope. To ensure concealment, one of the study staff was present at all evaluations and supervised that there were no discussions about the intervention between the blinded evaluators and the participants.

#### Study design, settings and participants

Study design: A two-arm, parallel-group, interventional randomized controlled trial was conducted at the Integral Health Clinic, Department of Physiology, All India Institute of Medical Sciences, New Delhi. This trial was completed between March 2017 and October 2019. The protocol of the study was approved by the Institutional Ethics Committee Board (reference number: IECPG371/29.06.2016) followed by registration in the Clinical Trial Registry of India (registration number: CTRI/2016/08/007136). The research was carried out following the Declaration of Helsinki and Good Clinical Practice criteria. Before starting the study, all participants sign a written informed consent form and provided a study information sheet. Each participant has to fill out a participant registration form which included all demographic data (education, marriage status, income, etc.) and as well as other physiological descriptions.

Inclusion and exclusion criteria: All the males and females 20–45 years of age who were recruited in the study are among those who visited the outpatient departments of our tertiary care institute through word-of-mouth, institute-related pamphlets, personal references, and referrals. All the suitable individuals were screened for eligibility to enroll in the study. The inclusion criteria were BMI 25 to 35 kg/m^2^, waist circumference of ≥ 90 cm in males, ≥ 80 cm in females, waist-hip ratio ≥ 0.9 for males, and ≥ 0.85 for females as per Asian Indian criteria for obesity (1). No history of participation in any lifestyle intervention whether or not it was for weight reduction in last 6 months, prior to their enrolment in the study.

The study protocol includes the following exclusion criteria: uncontrolled type 2 diabetes mellitus, and/or high blood pressure, other chronic inflammatory diseases diagnosed, severe psychiatric illness, regular use of non-steroidal anti-inflammatory drugs, current history of excessive alcohol consumption > 10 drinks/week for females and > 15 drinks/week for males (1 drink = 2 units of alcohol/1 unit equals 10 ml or 8 g of pure alcohol, the amount of alcohol an average adult can process in an hour) and daily smokers who were smoking at least 1 cigarette per day over the past month) (48), pregnant and lactating women, and women planning a pregnancy during the study period.

### Participants

**Figure 1:**
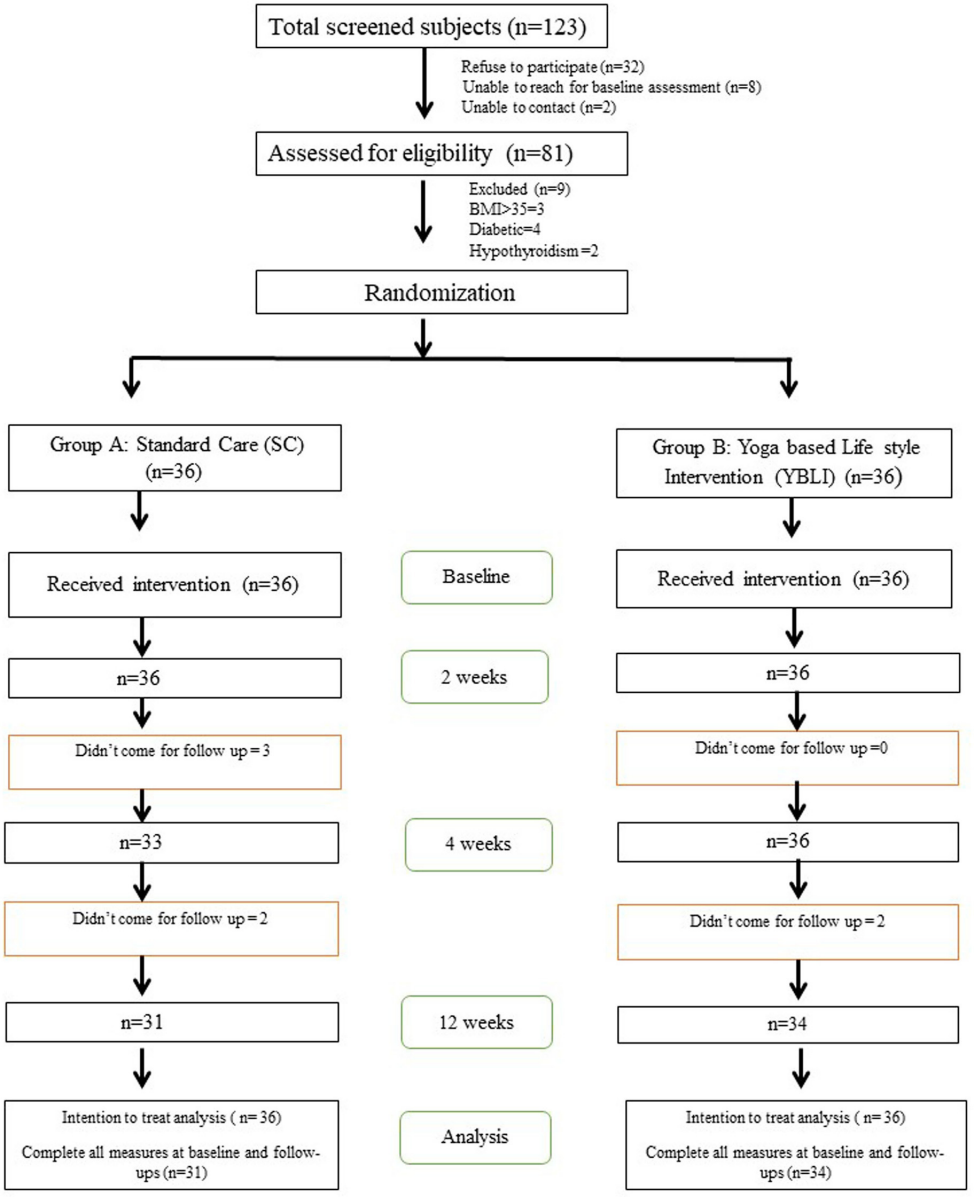
Study CONSORT flow diagram, consolidated standards of reporting trials, SC, Standard Care; YBLI, Yoga-based lifestyle intervention.

The participant disposition was according to the CONSORT guidelines is shown in Figure 1 total of 123 people were screened for obesity between March 2017 and October 2019, 72 (males = 21, females = 51) of whom were included and randomized into SC (*n* = 36) and YBLI (*n* = 36). The demographic components like age, sex, education, physical activity, nutrient indices (total energy, carbohydrate, protein, fat etc), anthropometric parameters like height, weight, body mass index, waist circumference, hip circumference, waist hip ratio and other physiological parameters like systolic and diastolic blood pressure as well as pulse rate were comparable between the two intervention groups at the beginning of the study (Table 1), also shown in our earlier publication associated with this study (29).

**Table 1:** Comparison of baseline characteristics of study participants.

**Demographic and physiological parameters**				
Age, years (mean ± SD)		37.3 ± 6.0	38.6 ± 6.7	0.99
Sex *n* (%)	Male	9 (25)	12 (33.4)	
	Female	27 (75.0)	24 (66.6)	0.85^¥^
Education *n* (%)	Intermediate	3 (8.3)	4 (11.1)	
	Graduate	27 (75)	25 (69.4)	0.23^¥^
	Post graduate	6 (16.6)	7 (19.4)	
Physical activity *n* (%)	Low	15 (41.6)	19 (52.7)	0.19^¥^
	Moderate	19 (52.7)	17 (47.2)
	High	2 (5.5)	0 (0)
Nutrient indices/composition/ content				
Total energy, Kcal/d		1920.7 ± 697.0	1850.0 ± 441.0	0.38
Carbohydrates (% of energy)		53.2 ± 6.24	52.3 ± 7.72	0.41
Protein (% of energy)		13.3 ± 3.25	12.2 ± 4.34	0.67
Fat (% of energy)		31.9 ± 6.72	30.4 ± 7.23	0.37
Fiber (g/d)		13.3 ± 5.65	14.1 ± 5.49	0.81
Height (cm)		157.8 ± 5.2	159.7 ± 7.6	0.22
Weight (kg)		78.0 ± 6.8	77.3 ± 9.5	0.77
Body mass index (kg/m^2^)		31.4 ± 3.3	30.7 ±3.2	0.36
Waist circumference (cm)		98.2 ± 8.3	98.4 ± 7.4	0.90
Hip circumference (cm)		107.2 ±7.0	108.0 ± 5.9	0.60
Waist hip ratio		0.91 ± 0.06	0.90 ± 0.06	0.43
Systolic blood pressure (mmHg)		125.4 ± 4.6	126.0 ± 11.6	0.79
Diastolic blood pressure (mmHg)		76.2 ± 7.8	77.8 ± 8.5	0.41
Pulse rate (bpm)		79.1 ± 8.8	79.9 ± 9.1	0.67

*Data are expressed as number (Percentage) n (%) for categorical data; mean ± standard deviation (SD); (p value obtained from independent t-test and from Chi square test^¥^)*.

*SC Standard Care; YBLI, Yoga-based Lifestyle Intervention*.

### Study interventions

#### Standard care (SC) for obesity

The standard care used in this study included an individualized specific diet plan and physical exercise. The participants were encouraged to engage in a 30-min light to moderate physical exercise, for at least 5 days a week. This could include brisk walking, stair climbing, and jogging (4-7 m/sec), (49). Each participant was provided individualized diet plan, based on their age, nutritional history, and food preferences, developed by a qualified dietician. The diet plan was thoroughly explained to each participant. Overall, the recommended diet included based on participants energy need, 50–60% by carbohydrates, <30% by fats, <10% saturated fat, <10% monounsaturated fatty acids, 5–8% polyunsaturated fatty acids, and 10–15%, calories by proteins. Also, it included cholesterol <200 mg/day, dietary fiber 25–40 g/day and salt intake <5 g/day. The diet was in-line with dietary guidelines for healthy living and obesity prevention and related disorders in Asian Indians (50).

#### Yoga based lifestyle intervention (YBLI)

**Figure 2:**
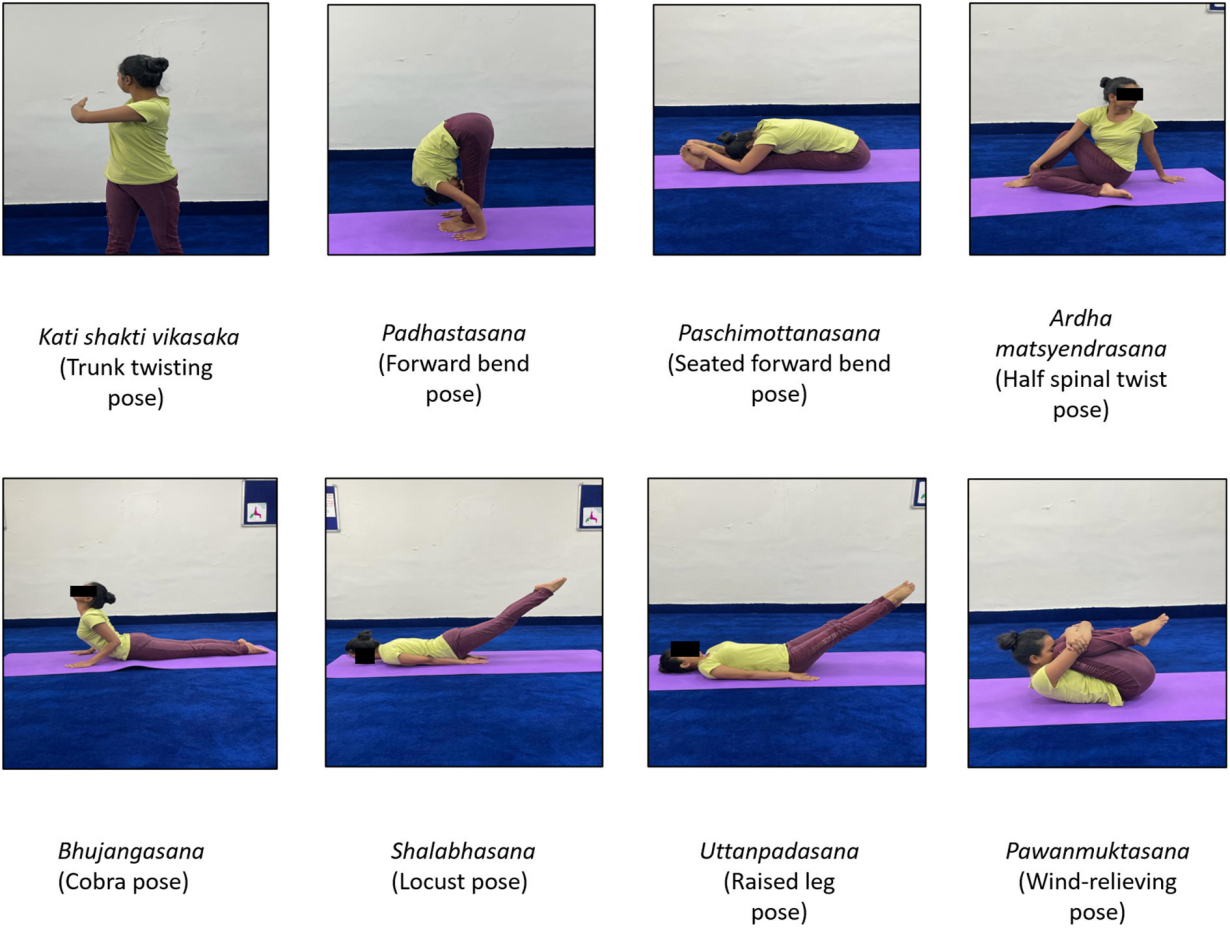
Images showing different Yoga-practice postures.

Individuals in the YBLI group directed a 12-week standardized, pretested yoga-based lifestyle intervention which is an integrated program consisting of theory and practice sessions (21–23, 26–29, 45). A typical day starts with a set of *asanas* (physical postures), *pranayama* (breathing exercises), *dhyana* (meditation) conducted by a trained yoga instructor for approximately 1 h to set a positive start of the day. The participants are then pulled in a mind engaging activity that orients them toward a yoga-based healthy lifestyle. This is delivered in the form of discussions/lectures on principles of yoga, yogic techniques, stress management, healthy diet, and eliminating elements of negativity (substance abuse etc.). The intervention is delivered to a group of six to eight participants at a time, allowing them ample time to interact with the experts (medical doctors, yoga experts, and dietitians). YBLI sessions were conducted 6 days a week of approx. 60 min duration/day for the first two weeks at the Integral Health Clinic (IHC) by a qualified and experienced yoga therapist with study investigators under the supervision of medical doctors. It was then a home-based practice (for the next 10 weeks). Details of the activities on a typical day during the YBLI are summarized in Table 2 and Figure 2.

**Table 2:** Details of activities performed in a single session of the Yoga-Based Lifestyle Intervention.

**S.No**.	**Practice to be done**	**Duration**
1.	Om Chanting	2 min
2.	Cleansing practice *Kapalbhati* (Skull Shining Breath)	5 min
3.	*Yogic Sukshma Vyayam* (Loosening exercise) Warm ups: starting from the head, working toward the toes 1. *Griva shakti vikasaka kriya* (Neck movements) 2. *Skandh chakra* (Shoulder rotation) 3. *Kati shakti vikasaka* (Trunk twisting pose) 4. *Jhanga shakti vikasaka* (Chair pose arms forward flow)	5 min
4.	*Suryanamaskara* (Sun salutation) (12 postures with slow and rhythmic breathing)	10 min
5.	Asanas: Standing postures; 1. Tadasana (mountain pose) 2. *Urdhva Hastottanasana* (upward salute side bend pose) 3. *Padhastasana* (forward bend pose) 4. *Ardha Chakrasana* (backward bend pose) 5. *Trikonasana* (triangle pose)	
6.	Sitting posture: 1. *Paschimottanasana* (seated forward bend pose) 2. *Ardha Matsyendrasana* (half spinal twist pose) 3. *Ushtrasana* (camel pose) 4. *Shashankasana* (hare pose)	25 min
7.	Prone: 1. *Viprit Naukasana* (reverse boat pose) 2. *Bhujangasana* (cobra pose) 3. *Shalabhasana* (locust pose)	
8.	Supine 1. *Uttanapadasana* (raised leg pose) 2. *Setubandhasana* (bridge pose) 3. *Pawanmuktasana* (wind relieving pose)	
9.	*Shavasana* (corpse pose)	5 min
10.	Pranayama (breathing practice/regulation) *Anulom Vilom* (alternate nostril breathing) *Bhastrika* (bellows breath) *Bhramari* (huming bee breath)	15 min
11.	Om Chanting	2 min

#### Compliance

To enhance the recommended YBLI and SC at home, participants in both groups were followed up regularly with phone calls/ WhatsApp as well as regular checking of their daily diary during the monthly visits at IHC up to 12 weeks from baseline. To ensure compliance with the intervention, participants received general nutritional advice and a personalized sample menu plan in writing and were advised during the follow-up examination. Participants in the YBLI group also received written instructions on yoga practice and, if necessary, provided yoga practice during the follow-up of the study. Participants were asked to create a formal daily exercise log at home to record compliance, time spent, and reasons. Compliance was assessed weekly by collecting and reviewing exercise records from home meals, physical activity, and yoga sessions as needed. Participants who did not comply with a particular week's data collection or partial documentation were treated as less compliant. Overall compliance with the intervention program and compliance with the SC group was (88%) and YBLI group (92%).

#### Study assessments

##### The primary objective

Estimation of Relative fold change in the expression of genes related to stress [Nuclear factor-kappa B (NF-Kappa B)], inflammation [Tumor necrosis factor-α (TNFα), interleukin-6 (IL-6)], and aging telomerase reverse transcriptase (TERT) in peripheral blood mononuclear cells using Quantitative Real-Time PCR Using SYBR GREEN Chemistry.

Fasting venous blood samples (5 mL) were collected from each participant at 8:45 am and immediately put in a vacutainer tube containing (K_2_) EDTA containing strong anticoagulant for separation of peripheral blood mononuclear cells.

This peripheral blood mononuclear cells after checking their viability through a hemocytometer and trypan blue dye were later stored in (RNA later stabilization solution, Cat. No: AM 7021, Invitrogen, California, USA) for the isolation of RNA from the cells.

Later, RNA was extracted using (QIAamp RNA Blood Mini Kit, Cat. No-52304, Qiagen, Hilden, Germany*)* following the manufacturer's instructions and the standard protocol. The eluted RNA (20 μl) was collected immediately, RNA quality was determined by taking OD 260/OD 280, and a ratio of ≥1.8 was considered to be good-quality RNA.

RNA was converted into cDNA by using i-script cDNA synthesis kit, Cat. No: 1708891 (BIO-RAD, California, United States) with 1 μg of RNA using the manufacturer's protocol and stored at −80°C till its further usage for estimation of relative fold changes in mRNA levels of stress (NF-Kappa B), inflammatory (TNFα, IL-6), and aging (TERT) genes by Quantitative real-time PCR (CFX96, BioRAD, California,United states).

In the present study, the target gene sequences are the genes of stress (NF-Kappa B), inflammation (TNFα, IL-6), and aging (TERT). One reaction is employed for a specific gene, where a single primer pair is present in a well. The CFX96 real–time system (Bio–Rad, CA, United States) quantified the relative gene expression using (Thermo Scientific, Massachusettes, USA, DyNAmo Flash SYBR Green qPCR Master Mix, Cat.No: F416L). The protocol for gene amplification was standardized at 35 cycles. Normalization of the amount of expressed mRNA used three internal housekeeping genes GAPDH, β-actin, and β-2 Microglobulin. The relative fold of gene expression was done by the 2^−Δ*ΔCt*^ method (51–53). Each cDNA product formed was tested in triplicate. The primer sequences were taken from OriGene Technologies (Rockville, MD) qPCR Primer Pairs, which are included in Table 3.

**Table 3:** List of oligonucleotides used in this study.

**Oligonucleotides/Gene**	**Primer sequence**
Tumor Necrosis Factor α (TNF-α)	Forward Primer-5′-CTCTTCTGCCTGCTGCACTTTG-3′ Reverse Primer- 5′-ATGGGCTACAGGCTTGTCACTC-3′
Interleukin−6 (IL-6)	Forward Primer- 5′-AGACAGCCACTCACCTCTTCAG-3′ Reverse Primer- 5′-TTCTGCCAGTGCCTCTTTGCTG-3′
Nuclear Factor Kappa B (NF Kappa-B)	Forward Primer- 5′-GCAGCACTACTTCTTGACCACC-3′ Reverse Primer- 5′-TCTGCTCCTGAGCATTGACGTC-3′
Human Telomerase Reverse transcriptase (TERT)	Forward Primer-5′-GCCGATTGTGAACATGGACTACG-3′ Reverse Primer- 5′-GCTCGTAGTTGAGCACGCTGAA-3′
Human Glyceraldehyde phosphate dehydrogenase (GAPDH) (Housekeeping gene used as an internal control)	Forward Primer-5′-GTCTCCTCTGACTTCAACAGCG-3′ Reverse Primer- 5′-ACCACCCTGTTGCTGTAGCCAA-3′
Human β- Actin (Housekeeping gene used as an internal control)	Forward Primer: 5′-CACCATTGGCAATGAGCGGTTC-3′ Reverse Primer: 5′-AGGTCTTTGCGGATGTCCACGT-3′
Human β-2 Microglobulin (Housekeeping gene used as an internal control)	Forward Primer: 5′- CCACTGAAAAAGATGAGTATGCCT-3′ Reverse Primer: 5′- CCAATCCAAATGCGGCATCTTCA-3′

### Baseline assessments

#### Anthropometric measurements

All anthropometric measurements were performed in minimal acceptable clothing and a fasting state without shoes. Calibrated electronic weighing scale (Seca 813, Germany) and wall-mounted stadiometer (Holtain Ltd, United Kingdom) were used to measure body weight and height to the nearest 0.1 kg and 0.1 cm respectively. BMI was calculated as weight in kilograms divided by height in meters squared. Waist circumference (WC) was measured midway between the lowest palpable rib and the top of the iliac crest, at the end of normal expiration using non-elastic measuring tape with a precision of 0.1 cm (Seca201, Germany). Hip circumference (HC) was measured in a standing posture at the point of maximum circumference over the buttocks. Waist to hip ratio (WHR) was calculated as waist circumference divided by hip circumference.

#### Measurement of blood pressure and pulse rate

Diastolic blood pressure, systolic blood pressure, and pulse rate were measured after 5–10 min rest in sitting posture. All measurements were performed with a validated semi-automatic blood pressure monitor (Citizen CH-432, Japan).

#### Dietary and physical activity assessment

A pretested, open-ended, semi-quantitative food frequency questionnaire was used to collect nutrient and dietary information on different food groups and miscellaneous food items from each participant at every visit (Appendix-I). For easy recollection of dietary intake, standardized serving bowls, spoons, and glasses of various sizes were demonstrated to the participants to estimate the portion sizes of the meal. Conversion of raw foodstuffs into nutrients was done by the software Diet Cal (version 8.0) programmed as per Indian food composition tables given by the Indian Council of Medical Research (54). International Physical Activity Questionnaire short form (IPAQ-SF) was used to assess physical activity levels in adults (55). It comprises 7 items used to record the activity of three intensity levels and idle hours. The IPAQ-SF estimates the overall physical activity level of an individual in Metabolic equivalent (MET)-min/week by determining the duration (in min) and frequency (times/day in a week). Data collected using IPAQ-SF was reported in categorical scores according to the guidelines for data processing and analysis of the IPAQ-SF. Levels of physical activity were categorized as follows:

***Category 1, low:*** This is the lowest level of physical activity. Those individuals who don't meet the criteria for categories 2 or 3 are considered low/inactive.
***Category 2, moderate:*** Any one of the following 3 criteria:
  a. 3 or More Days of Vigorous Activity of at Least 20 min per day or
  b. 5 or More Days of Moderate-Intensity Activity or Walking of at Least 30 min per day or
  c. 5 or More Days of any Combination of Walking, Moderate-Intensity, or Vigorous-Intensity Activities Achieving a Minimum of at Least 600 MET-Min/Week.
***Category 3, high:*** Any one of the following 2 criteria:
  a. Vigorous-intensity activity on at least 3 days and accumulating at least 1,500 MET-min/week or
  b. 7 or More Days of any Combinations of Walking, Moderate-Intensity, or Vigorous-Intensity Activities Achieving a Minimum of at Least 3000 MET-Min/Week.

The association between the IPAQ-SF and objective measures of activity or fitness in most of the studies shows a weak correlation still it is used as an assessment tool for physical activity worldwide (56).

#### Sample collection

During this 12-weeks study, the participants were evaluated for RNA fold changes at baseline and after 2, 4, and 12 weeks. The phlebotomists collected the samples in the morning between 8:45 am and 9:15 am at baseline as well as for the subsequent 2,4, and 12-weeks time points. Whole blood was used for isolation of peripheral blood mononuclear cells followed by RNA isolation. Immediately RNA was reverse transcribed into complementary DNA and then was stored at −80°C until further analyzed.

#### Study endpoints

The primary endpoint was to assess the RNA fold changes in NF-kappa B, TNF-α, IL-6, and TERT at 12 weeks between the two groups. Additional assessment time points were included at 2 and 4 weeks. The 2-week evaluation time point was included because the first two weeks of the YBLI were completed at Integral Health Clinic (IHC) under the direct supervision of a well-qualified and experienced yoga therapist and study investigators. Medical doctors constantly observed closely and interacted with the participants for the first two weeks at IHC. This allowed us to assess the effectiveness with good compliance, which was noted in our previous studies as well (21–24, 26, 27, 29, 45). Also, for better study compliance, we intentionally added one more time point at the 4^th^ week to evaluate the adherence of the participants to the recommended yoga program at their home based intervention.

#### Statistical analysis

The data were analyzed using IBM SPSS Version 26.0 statistical software. For all analyses, the intention to treat the (ITT) population was used. For continuous variables, mean and standard deviation (SD) is used, while for categorical data, number (%) is used. Between-group comparisons of categorical variables were performed using chi-square/Fisher's exact test. An independent *t*-test (for parametric data) was used to compare the continuous variables between the groups. RM-ANOVA was also used to assess within-group changes. Two-tailed tests were used in all of the tests. The intention-to-treat concept was used to conduct the analyses.

#### Consolidated standards of reporting trials (CONSORT) guidelines

The reporting of the trial has been done as per the Consolidated Standards of Reporting Trials (CONSORT) statement, which provides standard guidelines for reporting the results of a randomized trial (57).

#### Safety evaluation

As discussed earlier (29) safety guidelines were followed in both the intervention groups (58, 59) and safety was assessed throughout the study, though in our study there was a very minimal potential risk of adverse events. Adverse events (AE) were formally assessed during follow-up visits, and participants were requested to report any health-related events they encountered throughout and after the intervention to the yoga therapist.

Yoga postures were closely supervised by the experts, and have been pre-tested and standardized for obese individuals in our previous studies. Some of the participants experience muscle strain and soreness in the initial days of training and practice, which are mostly mild and resolve on their own (60). No serious adverse event was reported in our study, and none of the participants discontinued and no modifications to the yoga postures were required. AEs reported in the YBLI group were (11%) in which the most common ones are muscle strain and soreness, but this had no negative impact on their daily activities. AEs were not reported in the SC group.

#### Stopping rules or discontinuation criteria for individual participant

Participants withdrew if they did not physically attend the first two-week exercise program at Integral Health and Wellness Clinic, and were unable to practice yoga due to recent medical/surgical issues, became pregnant, or developed other conditions that prevented their participation. Discretion of treating physician.

## Results

Of all the 72 (100%) participants, 36 participants in each of the two groups completed the 2-week study and 31 participants (86%) in the SC group, and 34 participants (94%) in the YBLI group completed the 12-weeks study. A total of 7 participants dropped out of the study due to their relocation, health issues, prior commitments, and other personal reasons. The number of female participants in the study was higher, possibly because of the non-availability of the male individuals due to their jobs/professional preferences and commitment. The ITT analysis included all 36 participants from each group. There were no significant differences between the two randomly selected groups were detected at baseline (Table 1). In this study, most of the participants were engaged in low to moderate physical activity except for two participants who were engaged in high physical activity (Table 1). There were no notable reported adverse events that either deterred the participants from participation or impacted their day-to-day activities.

### Primary outcomes

The primary outcome was the relative fold change in mRNA of TNF-α, IL-6, NF-kappa B and TERT gene between the two groups at week 12, from them only TNF-α was significantly differ in SC and YBLI at week 12.

**Figure 3:**
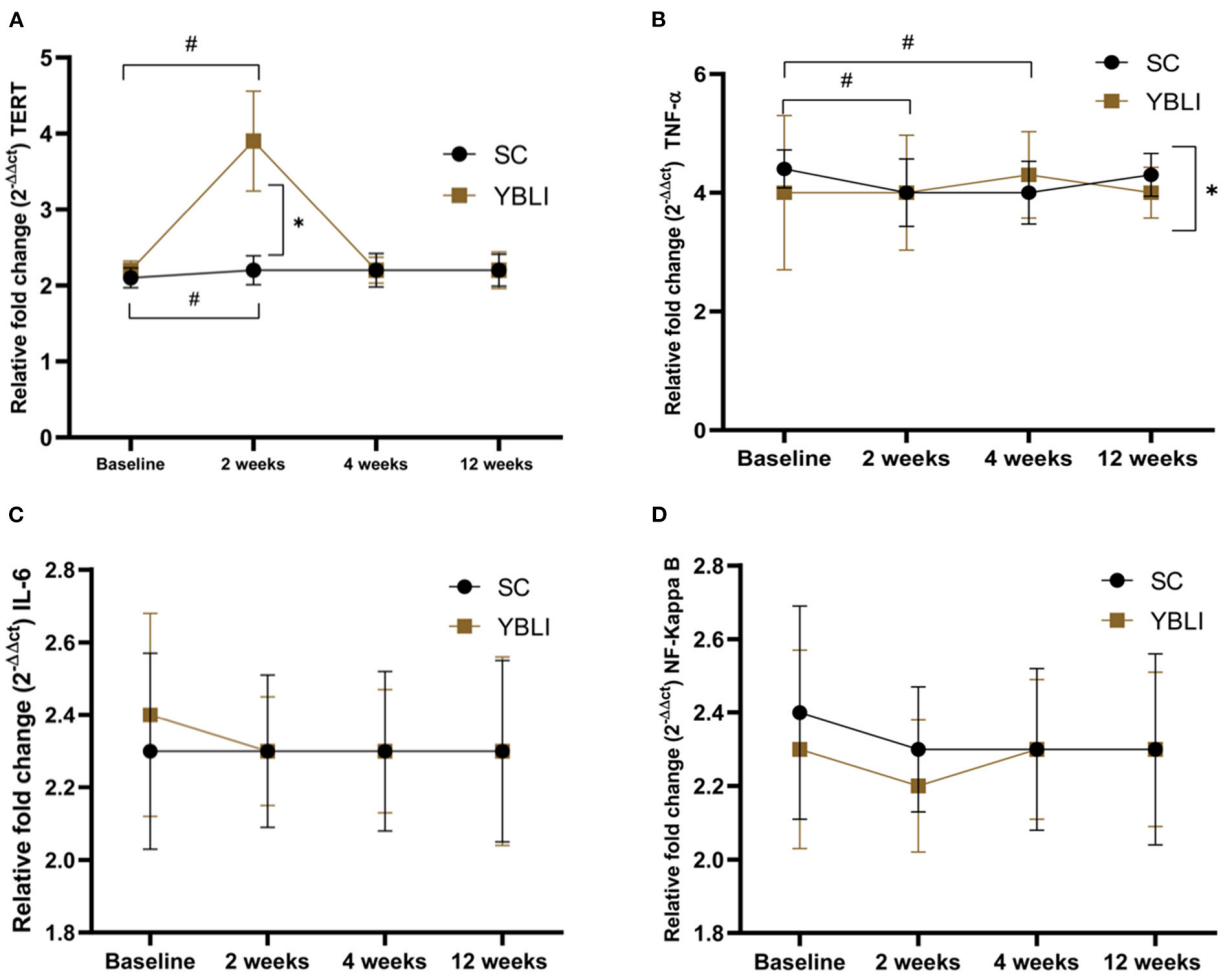
**(A)** Shows the relative fold change (2^−ΔΔct^) in the aging (TERT) gene between 2, 4, and 12 weeks of intervention in the Standard care (SC; *n* = 36) and the Yoga-based Lifestyle Intervention (YBLI; *n* = 36) groups. Data are expressed as mean ± SD. *p < 0.05 between the groups; #*p* < 0.05 from the baseline. **(B)** Shows the relative fold change (2^−ΔΔct^) in the inflammation (TNF-α) gene between 2, 4 and 12 weeks of intervention in the Standard care (SC; *n* = 36) and the Yoga-based Lifestyle Intervention (YBLI; *n* = 36) groups. Data are expressed as mean ± SD. *p < 0.05 between the groups; #*p* < 0.05 from the baseline. **(C)** Shows the relative fold change (2^−ΔΔct^) in the inflammation (IL-6) gene between 2, 4 and 12 weeks of intervention in the Standard care (SC; *n* = 36) and the Yoga-based Lifestyle Intervention (YBLI; *n* = 36) groups. Data are expressed as mean ± SD. **(D)** Shows the relative fold change (2^−ΔΔct^) in the oxidative stress (NF-Kappa B) gene between 2, 4 and 12 weeks of intervention in the Standard care (SC; *n* = 36) and the Yoga-based Lifestyle Intervention (YBLI; *n* = 36) groups. Data are expressed as mean ± SD.

The relative fold change (2^−ΔΔct^ values) of oxidative stress (NF-Kappa B) inflammation (TNF-α, IL-6) and aging (TERT) gene at 2, 4, and 12 weeks of intervention are summarized in Tables 4, 5 and Figures 3A–D. Within-group analysis for both the SC and YBLI groups were performed with data from week 2, week 4, and week 12, compared in each case to baseline data. Within YBLI and SC group TERT fold change was significantly increased after 2 weeks from the baseline (Table 4, Figure 3A). No significant change was evident at week 4 and week 12 for TERT gene in any of the groups (Table 4, Figure 3A). Within the SC group, TNF-α fold change was significantly reduced after 2 and 4 weeks but not at 12 week from the baseline (Table 4, Figure 3B), however in YBLI no such significant change was observed at any time points for TNF-α gene (Table 4, Figure 3B). Within-group analysis for NF-Kappa B and IL-6 both did not show any significant change at any time point in any of the groups (Table 4, Figures 3C,D).

**Table 4:** Relative fold change (2-^ΔΔct^ values) of oxidative stress (NF-Kappa B), Inflammation (TNF-α, IL-6) and aging (TERT) gene between at 2, 4, and 12 weeks of intervention in the SC (*n* = 36) and YBLI (*n* = 36), within groups comparison.

**Variable**		**Baseline**	**2 weeks** **(Mean ±SD)**	**4 weeks**	**12 weeks**	***p*** **-value week 2 vs baseline**	***p*** **-value week 4 vs baseline**	***p*** **-value week 12 vs baseline**
NF-Kappa B (2^−ΔΔct^)	SC	2.4 ± 0.29	2.3 ± 0.17	2.3 ± 0.22	2.3 ± 0.26	0.375	0.805	0.430
	YBLI	2.3 ± 0.27	2.2 ± 0.18	2.3 ± 0.19	2.3 ± 0.21	0.210	0.731	0.200
TNF-α (2^−ΔΔct^)	SC	4.4 ± 0.32	4.0 ± 0.57	4.0 ± 0.53	4.3 ± 0.36	0.004^**^	0.002^**^	0.098
	YBLI	4.0 ± 1.3	4.0 ± 0.97	4.3 ± 0.73	4.0 ± 0.43	0.942	0.218	0.842
IL-6 (2^−ΔΔct^)	SC	2.3 ± 0.27	2.3 ± 0.21	2.3 ± 0.22	2.3 ± 0.25	0.876	0.774	0.474
	YBLI	2.4 ± 0.28	2.3 ± 0.15	2.3 ± 0.17	2.3 ± 0.26	0.113	0.093	0.160
TERT (2^−ΔΔct^)	SC	2.1 ± 0.13	2.2 ± 0.19	2.2 ± 0.22	2.2 ± 0.21	0.036^*^	0.635	0.713
	YBLI	2.2 ± 0.12	3.9 ± 0.66	2.2 ± 0.17	2.2 ± 0.24	<0.0001^***^	0.168	0.744

*P-value for within-group change obtained by RM-ANOVA*.

*Bold values show the p-value showing the significant change*.

* *p < 0.05,*

** *p < 0.01, and*

*** *p < 0.001*.

**Table 5:** Relative fold change (2^−ΔΔct^ values) of oxidative stress (NF-Kappa B) inflammation (TNF-α, IL-6) and aging (TERT) gene between the SC (*n* = 36) and YBLI (*n* = 36) groups, between groups comparison.

**Variable**		**Baseline**	**2 weeks (Mean ±SD)**	**4 weeks**	**12 weeks**	***p*** **-value between groups at week 2**	***p*** **-value between groups at week 4**	***p*** **-value between groups at week 12**
NF-Kappa B (2^−ΔΔct^)	SC	2.4 ± 0.29	2.3 ± 0.17	2.3 ± 0.22	2.3 ± 0.26	0.32	0.65	0.52
	YBLI	2.3 ± 0.27	2.2 ± 0.18	2.3 ± 0.19	2.3 ± 0.21	
TNF-α (2^−ΔΔct^)	SC	4.4 ± 0.32	4.0 ± 0.57	4.0 ± 0.53	4.3 ± 0.36	0.92	0.053	0.0065^**^
	YBLI	4.0 ± 1.3	4.0 ± 0.97	4.3 ± 0.73	4.0 ± 0.43	
IL-6 (2^−ΔΔct^)	SC	2.3 ± 0.27	2.3 ± 0.21	2.3 ± 0.22	2.3 ± 0.25	0.40	0.44	1.00
	YBLI	2.4 ± 0.28	2.3 ± 0.15	2.3 ± 0.17	2.3 ± 0.26	
TERT (2^−ΔΔct^)	SC	2.1 ± 0.13	2.2 ± 0.19	2.2 ± 0.22	2.2 ± 0.21	**<0.0001** ^***^	0.58	0.836
	YBLI	2.2 ± 0.12	3.9 ± 0.66	2.2 ± 0.17	2.2 ± 0.2	

*P-Value for between-group change obtained from independent t-test*.

*Bold values show the p-value showing the significant change*.

*p < 0.05,

** *p < 0.01, and*

*** *p < 0.001*.

Between the two groups, relative fold change in the aging gene (TERT) was significantly higher in the YBLI group at 2 weeks only not at 4- and 12-weeks time points (Table 5, Figure 3A). Relative fold change in the inflammation gene (TNF-α) was significantly higher in the SC group at 12 weeks only but not at 2- and 4-weeks time points (Table 5, Figure 3B). The between-group analysis for NF-Kappa B and IL-6 did not show any significant difference in both the YBLI and SC groups (Table 5, Figures 3C,D).

## Discussion

Most of the previous studies published on yoga and gene expression had long-term practitioners in their studies or else they provided the intervention for the duration of 12 or more weeks. In our study, we evaluate the efficacy of YBLI (yoga + diet) over SC (physical exercise + diet) (for the 12 weeks with additional assessment time points on weeks 2 and 4, on oxidative stress, inflammation, and aging-specific gene expression. Here, we observed that both SC and YBLI might influence body physiology at the molecular level. Today's sedentary lifestyle is associated with physiological and psychological stress which is also influenced by various environmental cues and nutritional factors that accelerate the stress and affect us physically or mentally (61). Oxidative stress also accelerates cellular aging by affecting the various DNA repair mechanism as well as the telomere length of the chromosomes affects overall genomic stability (62). Adipose tissue in obese individuals seems to alter the expression of various stress and inflammatory genes like tumor necrosis factor α (TNF-α), Interleukin-6 (IL-6). The elevated level of oxidative stress in high BMI conditions is marked by the higher expression of NF-kappa B (63). The TERT gene encodes for an enzyme called telomerase. Telomerase maintains the chromosomal ends called telomeres, the shorter telomeres in the obese condition are correlated with accelerated cellular aging. The authors did not observe any significant difference between the two groups for NF-kappa B, IL-6, and TERT at week-12. Inflammatory gene marker TNF-α showed a significant difference in its fold change between the two groups at week-12. However, the interpretation and explanation of this result is difficult as the TNF-α level showed large fluctuations during the study period, being significantly decreased at the intermediate test points of weeks 2 and 4 and significantly increased at week 12 in the SC group. However, the aging gene marker TERT expression was significantly increased in YBLI versus the SC group at week 2. Additionally, within-group TERT expression was significantly increased in both SC and YBLI groups at the week-2 time point but did not show the same observation at week 4 and 12-time points. There were no other significant differences between the two groups at any other time point.

In our study, the relative fold change of aging-specific gene TERT was significantly higher in the YBLI group at 2 weeks. Our finding is supported by another study in which the aging-related TERT gene showed a higher trend in *Sudarshan Kriya* practitioners, who were practicing this form of yoga for the past year, however, in our study, we have observed a significant increase in the TERT gene expression after 2 weeks in both SC and YBLI groups though the increase was much higher in YBLI group (31). Another study conducted on hypertension patients who participated in Transcendental Meditation for 16 weeks showed a non-significant increase in the TERT gene expression after the intervention program (41), however, in our study, we did not observe this change for the TERT gene at week 12 in YBLI but it gives the impression that 12 or more weeks of yoga intervention might change the TERT expression. Similar to our study SC group (physical exercise+ diet), the 12-weeks resistance training program did influence the TERT expression in sedentary middle-aged adults (64). Cytokines such as tumor necrosis factor-α (TNF-α) and interleukin-6 (IL-6) play a major role in the body's inflammatory response. The effect of yoga-based lifestyle intervention on inflammatory markers like TNF-α and IL-6 has been investigated in several studies. The present study observed that between the two groups YBLI did not lead to a significant change in relative fold change of the inflammatory marker gene IL-6. However, the fold change of another inflammatory gene TNF-α was showing fluctuations and it significantly changed in the SC group at 2 and 4-week time points without modifying the IL- 6 at any time points. For IL-6 maybe in future studies, we should check the changes in abdominal fat content as it is correlated with abdominal fat deposition (65). It seems like inflammatory genes were not responding as they were intended to like in another similar study, in which 8 weeks of yoga-based lifestyle intervention significantly decreased the TNF-α and IL-6 expression in patients with rheumatoid arthritis (42). However, in our study fold change of IL-6 was remain unchanged in both the SC and YBLI groups. The reason behind this observation is poorly understood maybe intervention of more than 12 weeks will be required for modification of the IL-6. In another study, 12 weeks of aerobic exercises did not change the expression of the TNF-α gene in women with metabolic syndrome (66). Though in our study SC significantly decreases the TNF-α-fold change at 2 and 4 weeks, it was not steady and consistent till week 12.

NF-Kappa B is a possible link between oxidative stress, inflammation, and aging (67). The present study observed that YBLI did not lead to a significant change in relative fold change of NF-Kappa B when compared with standard care. The data of this study was found to be consistent with the recent RCT study in which 8 weeks of yoga-based lifestyle intervention was given to rheumatoid arthritis patients and found that expression of the NF- Kappa B gene was not significantly changed in the yoga group (42).

However, another study showed the conflicting result in which 8 weeks of *kirtan kriya meditation* significantly reduced the NF-Kappa B gene expression in dementia caregivers (38). A similar kind of observation was perceived in another study in which 12 weeks of Iyenger yoga intervention significantly reduced the NF-Kappa B expression in breast cancer survivors, (39). In tumors with high NF-Kappa B activity, the accumulation of pro-inflammatory cytokines in the tumor environment promotes tumor growth, therefore a slight reduction in NF-kappa B might have been showing a significant change and could be easily detected which we did not observe in our study.

Previous studies showed that YBLI was effective in positively modifying cellular aging in obese adults at the genetic level (29) and reducing oxidative stress and inflammation at the serum protein level (24, 45). Though YBLI might not have been showing a reliable significant change in the oxidative stress and inflammation markers at the RNA level, still there was a possibility that it might have increased the expression of aging specific TERT gene and helped slow down the aging process in Indian adults with obesity.

## Limitations of the study

The study had a few limitations like the absence of genome-wide expression analysis which gives more information about the large number of genes modified by the SC and YBLI. The Asian Indian criteria to define obesity were used therefore, the findings of the present study cannot be generalized to other population groups. The sample size was relatively small, and this could be a reason that the significant changes in both groups could not be achieved at week 12. The first 2-weeks intervention at Integral Health Clinic was more intense in the YBLI group than in the SC group which might have influenced the results during the first two weeks which were not observed later.

### Strength of the study

This RCT was the first study one of its kind that compares the two types of intervention groups, SC (physical exercise + diet) and YBLI (yoga + diet) in the Indian obese population. The aim was to assess the overall comparative effectiveness and benefits of the two interventions in modulating oxidative stress, inflammation, and aging per se at the transcriptional level.

### Future implications

Stress, inflammation, and aging have always been a concern, especially for those who are overweight or obese. The data suggest that there is a way to modulate inflammation and aging at the transcription level. But what could be the duration and intensity of the yoga components that are crucial for the transcriptomic changes need to be answered in future studies.

## Conclusion

Our results suggest that SC (physical activity and diet) itself, and in combination with yoga may have beneficial effects on inflammatory and aging processes in obese people. Continued RCT studies with a larger number of participants, and a possibly longer period of interventions, should focus on the detection of the change in the relative expression of TNF-α and TERT genes.

## Data availability statement

The original contributions presented in the study are included in the article/supplementary material, further inquiries can be directed to the corresponding author/s.

## Ethics statement

The studies involving human participants were reviewed and approved by Institute Ethics Committee, AIIMS, New Delhi, India. The patients/participants provided their written informed consent to participate in this study.

## Author contributions

PS: implemented trial, acquired data, performed the experiments, data analysis and interpretation, literature search, manuscript writing, and reviewing. RY: conceived the study, study design, data interpretation, analysis, manuscript writing, and reviewing. RK: study design and subject recruitment, manuscript writing, and reviewing. RD: study design and laboratory procedures, manuscript writing, and reviewing. All authors contributed to the article and approved the submitted version.

## Funding

Authors are grateful to the All India Institute of Medical Sciences, New Delhi, the Indian Council of Medical Research, Government of India, and Department of Science and Technology, SATYAM, Government of India for providing financial support.

## Conflict of interest

The authors declare that the research was conducted in the absence of any commercial or financial relationships that could be construed as a potential conflict of interest.

## Publisher's note

All claims expressed in this article are solely those of the authors and do not necessarily represent those of their affiliated organizations, or those of the publisher, the editors and the reviewers. Any product that may be evaluated in this article, or claim that may be made by its manufacturer, is not guaranteed or endorsed by the publisher.
